# Definition of a disability weight for human exposure to ionizing radiation and its application to the justification of medical exposure

**DOI:** 10.1093/bjro/tzae043

**Published:** 2024-12-18

**Authors:** Colin John Kotre

**Affiliations:** Institute of Health, University of Cumbria, Bowerham Road, Lancaster, LA1 3JD, United Kingdom

**Keywords:** radiation, detriment, DALY, justification, radiology

## Abstract

**Objectives:**

To establish a link between radiation dosimetry and disability-adjusted life-years (DALY) with the aim of quantifying the justification of medical exposures.

**Methods:**

The health detriment, defined as lifetime loss of DALY at age of exposure to ionizing radiation for a US-European population was calculated. A simple model of the relationship was fitted to the results. Apart from in late life within the latency period for radiation-induced cancers, most of the relationship can be adequately fitted to a straight line of negative gradient. The gradient of this line corresponds to a loss of DALY per year following exposure to radiation and is therefore equivalent to a disability weight (DW) used in the calculation of DALY.

**Results:**

Radiation dose-dependent DWs for radiation exposure to a US-European population are estimated as 0.020 DALY/yr/Sv for males and 0.022 DALY/yr/Sv for females.

**Conclusions:**

By comparing a range of 66 radiological examinations in terms of the DWs of the disease or injury states with the DWs resulting from the associated radiological exposures, it is demonstrated graphically that the resulting benefit is far greater than the detriment in every case.

**Advances in knowledge:**

The definition of a DW for ionizing radiation, proportional to effective dose as currently defined, can link radiation exposure to the existing large body of data on the DALY burden and DWs for a wide range of diseases and injuries, providing a means for the quantitative justification of the benefit-detriment balance of medical exposures.

## Introduction

In a previous paper,^1^ the results of a calculation of loss of disability-adjusted life-years (DALY) due to ionizing radiation exposure for a Japanese population^2^ were used to suggest that there exists an approximately linear loss of DALY with time after exposure. The negative slope of this line can be taken, under the present regulatory assumption of a linear-no-threshold relationship between radiation dose and radiation detriment,^3^ to be proportional to the whole-body radiation dose to the individual and has the units of DALY per year. This unit is the same as that defined for the disability weight (DW) used in the calculation of DALY and detailed below, and this finding suggests a method for linking the detriment resulting from radiation exposure to the large body of data (particularly from the World Health Organisation Global Burden of Disease [GBD] project) on DW and DALY loss for a wide range of diseases and injuries. The objective of this work is to establish a link between effective dose, the established measure of human exposure to ionizing radiation, and DALY, the established measure of health detriment in populations and the individual.

The work by Shimada and Kai^2^ and others^4^^,^^5^ is part of a discussion within the International Commission on Radiation Protection (ICRP) about a possible redefinition of the detriment aspects of effective dose in terms of DALY, but there are no firm plans for this change and alternative approaches may be adopted instead. In this paper, an illustrative calculation for the lifetime loss of DALY with age at exposure is performed for a US-European population (male and female) using radiation-induced cancer incidence data derived using the present ICRP Report 103 definition of effective dose.^3^ The results from this calculation and from the subsequent evaluation of DWs for radiation exposure should therefore be applicable within the current system of radiation protection.

### Disability-adjusted life-years

The DALY quantifies the impact of a disease on a population by combining mortality and morbidity into a single metric. The DALY is defined as^6^:

(1) $$\mathrm{DALY}=\mathrm{YLL}+\mathrm{YLD}=N_{m}.LE+N_{i}.DW.YD$$

where DALY = disability-adjusted life year; YLL = years of life lost due to premature mortality (year); YLD = years lived with a disability (year); N_m_ = number of deaths (person); LE = standard life expectancy at age of death (year/person); N_i_ = number of incident cases (person); DW = disability weight (DALY/year); YD = mean years of disability (year/person).

The result of this calculation is an estimate of the number of years of healthy life lost to premature death and disability due to disease or injury in a population. Of particular importance in relation to the problem of assessing benefit and detriment from radiological procedures is the disability weight, DW (referred to as the health state weight in some WHO documents^6^) The DW represents a rate of health loss as the fractional number of healthy years lost per year of disability. The values of DW range from 0 (perfect health: no loss) to 1 (dead) and have been established using large-scale international surveys to elicit judgements on the health losses associated with causes of disease and injury. More than 30 000 such surveys have been conducted. Strong evidence for consistent results across samples from different cultures has been reported.^7^ The approach taken in this work will be to estimate a dimensionless number representing the ratio between the individual lifetime DALY gained by undergoing a successful medical radiological procedure, and the individual lifetime DALY lost due to the associated exposure to ionizing radiation. This number will be used as a quantitative measure of justification in terms of the ratio of benefit to detriment.

## Methods

### Calculation of lifetime DALY loss with age at exposure for a US-European population

The calculation was based on the approach of Vaillant et al,^4^ who derived an approximate result for individual DALY per Sv for a general population by multiplying the radiation-induced cancer and heritable effects incidence rates from ICRP Report 103^3^ by a lifetime factor of DALY per incidence for the various cancers considered to be radiation-induced, and DALY per incidence for heritable effects. The starting point for the present calculation was the age- and sex-specific radiation-induced cancer incidence rates for a US-European population given by Wall et al.^8^ These data are presented in 10-year age bands and are derived using the definitions and models of ICRP Report 103.^3^ The values of DALY per incidence for male and female were calculated in 5-year age bands from DALY and incidence data given in the large GBD data resource available online.^9^ The final values of lifetime DALY loss at age of exposure were obtained by multiplying the radiation-induced cancer incidence rates at the time of exposure^8^ by the population age-banded DALY integrated over the remaining lifetime of the individual divided by the population age-banded incidence also integrated over the remaining lifetime of the individual. A 5-year minimum latency period for solid cancers and a 2-year minimum latency period for leukaemia was allowed in line with the approach taken by Shimada and Kai.^2^

For increased consistency with the incidence data in Wall et al,^8^ and with data in ICRP Report 103,^3^ the DALY and incidence data were taken from the year 2000. DALY and incidence data for each cancer type were obtained for the US and WHO Europe regions separately and then combined using a population-weighted average in each age band for male and female. This calculation was done in 5-year age bands to allow a more accurate inclusion of the latency periods in the integration over lifetime following exposure.

For leukaemia associated with the absorbed dose in red bone marrow, DALY and incidence values for chronic lymphocytic leukaemia were subtracted from the values for all leukaemia in line with the approach taken in ICRP Report 103.^3^ For liver cancer, the “other causes” category was used to eliminate other known mechanisms of liver cancer induction (hepatitis B, hepatitis C, alcohol use, nonalcoholic steatohepatitis). For “other solid cancers”, the group of solid cancers not already included but contributing most to DALY detriment to the population were identified. Such cancers were included if their rate of DALY loss per 10 000 was greater than 100. For males, these were cancers of the larynx, prostate, lip and oral, pancreas, kidney, brain, and “other malignant”. For females, these were cancers of the pancreas, brain, cervix, uterus and “other malignant”. The ratios of DALY loss to incidence were averaged within each of the 2 groups of cancers.

The radiation-related cancer incidence data from Wall et al^8^ do not include heritable effects associated with exposure of the gonads. ICRP Report 103^3^ does, however, quote age-averaged risk coefficients of 20 cases per 10 000 per Sv for ages 0-85 at exposure male and female, and 12 cases per 10 000 per Sv for ages 18-64 at exposure male and female. From these figures, a simple monotonic function of cases per 10 000 against age band can be derived that has an average value of 20 for ages 0-85 and 12 for ages 18-64. This is shown in the second column of Table 1. In order to produce incidence figures in 10-year age bands it is necessary to consider the population age distribution in the age bands so that the incidence figures are per 10 000 in each age band, rather than a flat average over the whole population. Suitably corrected figures based on the averaged US-European population age distributions for male and female (year 2000) are shown in the third and fourth columns of Table 1. Since only lifetime DALY loss and incidence at birth are required to approximately quantify the detriment of heritable effects, age banding for these effects was not used, and the same value was applied to both sexes. Vaillant et al^4^ suggest a figure of 43.1 DALY per incidence, based on global figures from Huijbregts et al^10^ and a population-weighted average figure of 43.1 was adopted for the present calculation. Attempts to derive a figure from the GBD data^9^ were hampered by a lack of incidence information for some heritable effects.

**Table 1.** A simple monotonic function of incidence for heritable effects against age which gives the same average values for all ages, and for working age (18-64) as are given in ICRP Report 103.^3^ Column 2 is the function for a flat age distribution, and columns 3 and 4 show the function adjusted for the male and female population distributions to give the similar age-weighted averages.

Age	Incidence function for heritable effects (per 10 000)	Male population-weighted incidence for heritable effects (per 10 000)	Female population-weighted incidence for heritable effects (per 10 000)
0-9	100	80	86
10-19	50	37	42
20-29	26	18	19
30-39	13	8	9
40-49	7	4	4
50-59	3	2	2
60-69	1	1	1
70-79	0	0	0
80-89	0	0	0
90-99	0	0	0

	Averages:	Male age-weighted averages:	Female age-weighted averages:
	Age 0-99 = 20.0	Age 0-99 = 20.11	Age 0-99 = 19.95
	Age 18-64 = 11.91	Age 18-64 = 12.18	Age 18-64 = 12.12

Abbreviation: ICRP = International Commission on Radiation Protection.

Table 2 shows a comparison between the DALY per incidence coefficients used by Vaillant et al^4^ and the coefficients being used in the present calculation averaged (with population weighting) across age and sex. The two would not be expected to be identical as these are for different populations. One obvious difference can be accounted for in that Vaillant et al, without a direct figure for thyroid, uses an average figure from other organs, whereas thyroid data is used directly here. Some disagreement can be seen for liver cancer and leukaemia and it is perhaps notable that the coefficients for both were derived here from specific subsets of the DALY and incidence data (as above). Despite these differences, the general pattern of agreement suggests that the methodology being adopted here is appropriate.

**Table 2.** Comparison between the values of DALY per incidence used by Vaillant et al^4^ and an indicative age-weighted averaged and sex-averaged summary value for the age- and sex-specific values used in this work. Differences are explained in the text.

Organ/tissue	DALY per incidence (Vaillant et al^4^)	Age-weighted and sex-averaged DALY per incidence (this work)
Oesophagus	17.9	16.0
Stomach	13.6	13.9
Colon	8.8	9.0
Liver	22.5	16.4
Lung	16.5	15.8
Breast	7.6	8.1
Ovary	13.3	15.2
Bladder	5	8.1
Thyroid	13.35	6.8
Bone marrow	28.3	9.3
Other solid cancer	11.5	11.6
Gonads (heritable)	43.1	43.1

Abbreviation: DALY = disability-adjusted life-years.

**Figure 1.**
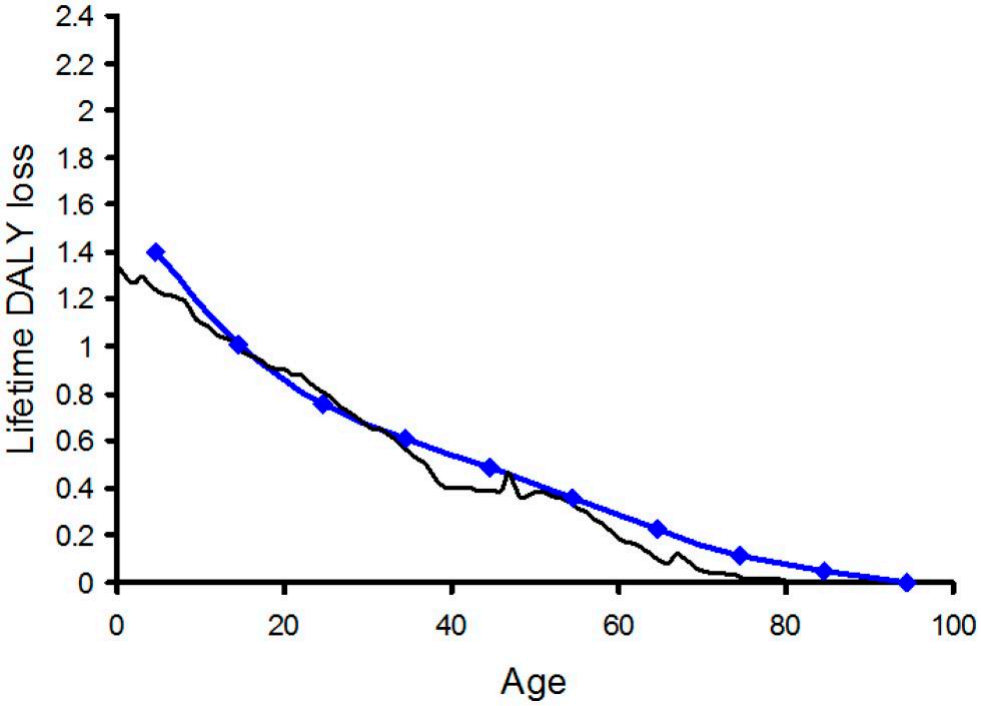
The calculated lifetime loss of disability-adjusted life-years at age of exposure to 1 Sv for US-European males (coloured line). This is compared with the result for Japanese males transcribed from reference 2 (black line).

**Figure 2.**
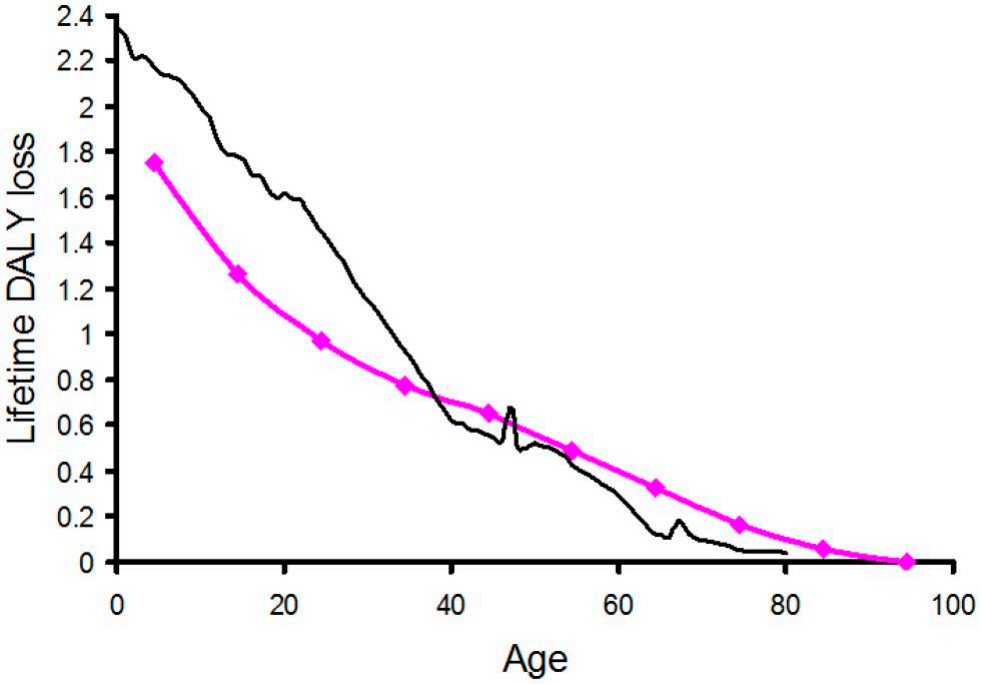
The calculated lifetime loss of disability-adjusted life-years at age of exposure to 1 Sv for US-European females (coloured line). This is compared with the result for Japanese females transcribed from reference 2 (black line).

Figures 1 and 2 show the calculated lifetime DALY loss in a US-European population plotted against age at exposure to an effective dose of 1 Sv for male and female, respectively. The results are plotted as bold coloured lines and are compared against the transcribed results of Shimada and Kai^2^ for a Japanese population. The shape of the curves is similar, although differences would be expected due to the differing methodologies and data sources, together with the greater longevity of the Japanese population which is particularly pronounced in females. The form of both the male and female plots suggests a reasonable fit to a constant negative rate of change over most of the age range followed by smaller rates of change at later ages. This is consistent with a continuing detriment after radiation exposure due to the latency periods for the expression of radiation-induced cancers. The latency period for radiation-induced leukaemia is thought to extend from a minimum of about 2 years to a peak at around 7 years. At around 25 years few further cases are seen. For radiation-induced solid cancers, the minimum latency period is thought to be 5-10 years peaking at approximately 40 years, with cancers continuing to appear in an exposed population long after that.^2^^,^^11^^,^^12^ Analysis of the atomic bomb survivor data has shown that the excess absolute rate of radiation-induced cancer occurrence increases with age, suggesting that the excess risk persists over a lifetime.^12^ The downward slopes of Figures 1 and 2 show this with a population exposed at a younger age exhibiting a greater lifetime DALY loss because they have lived with the continuing detriment from the exposure for longer. Conversely, for an older population containing an increased proportion of members with survival times less than the latency period for radiation-induced cancers, the cancer induction risk falls to close to zero because there is not enough remaining lifetime for many cancers to appear, and the gradients of the plots flatten out.

### Derivation of a disability weight for exposure to ionizing radiation

**Figure 3.**
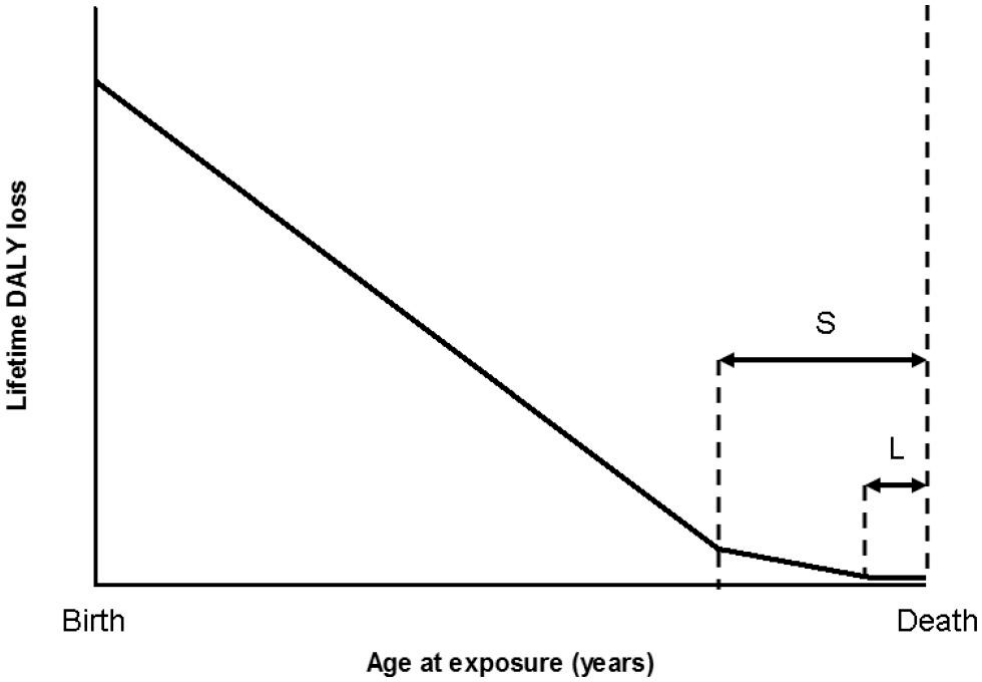
The lifetime loss of disability-adjusted life-years for an individual modelled as a linear decrease with age at acute exposure up to an age where the individual is within the solid cancer latency period of their death, S, followed by a smaller gradient due to leukaemia up to the shorter latency period from death for this disease, L, then a low residual value for the rest of their life.

**Figure 4.**
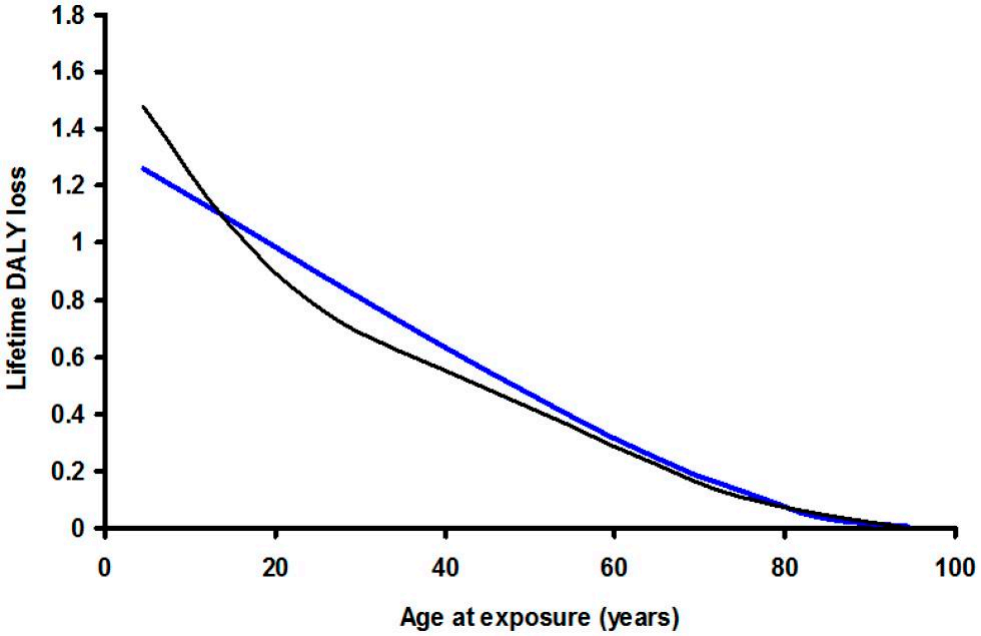
The hypothetical model for individual lifetime loss of disability-adjusted life-years from Figure 3 fitted to the calculated results for a US-European male population using US^13^ and European^14^ life tables to predict the age of death for each point plotted.

In Figure 3, the lifetime loss of DALY for an individual is modelled as a linear decrease with age at acute exposure at a rate R up to an age where the individual is within the solid cancer latency period of their death, S, followed by a smaller gradient due to leukaemia up to the shorter latency period from death for this disease, L, then a low residual value for the rest of their life.^1^ This model was fitted to the results of Figures 1 and 2. The minimum latency period for solid cancers of 5 years, and that for leukaemia of 2 years were inserted into the model in line with the calculation above, leaving R as the only fitted variable. The rate of change of DALY per year in the leukaemia-only portion of the model was set in proportion to R from the results of the calculation above, with the leukaemia DALY loss set at 11.7% of the total in males and 5.3% in females. This compares with the results of Shimada and Kai^2^ who found the leukaemia DALY loss to be 13% of the total in males and 7.1% in females. At each age plotted, population-averaged US^13^ and European^14^ life tables (year 2000/1999) were used to estimate the age of death. Working back through the latency period for leukaemia where the DALY loss is close to zero, the rate of change for leukaemia alone was applied up to the end of the latency period for solid cancers, after which the R rate of change was applied back to the age being considered, and the point plotted. The root mean squared error between the model and the data of Figures 1 and 2 was minimized by adjusting the value of R. Figure 3 shows the model plotted against the original data for males and Figure 4 for females. The best fit for males was obtained with a value for R of −0.020 DALY per year for an exposure of 1 Sv. The best fit for females was obtained with a value for R of −0.022 DALY per year for an exposure of 1 Sv. (The values of R previously fitted to Japanese population data were −0.019 DALY per year for males and −0.030 DALY per year for females for an exposure of 1 Sv.^1^)

**Figure 5.**
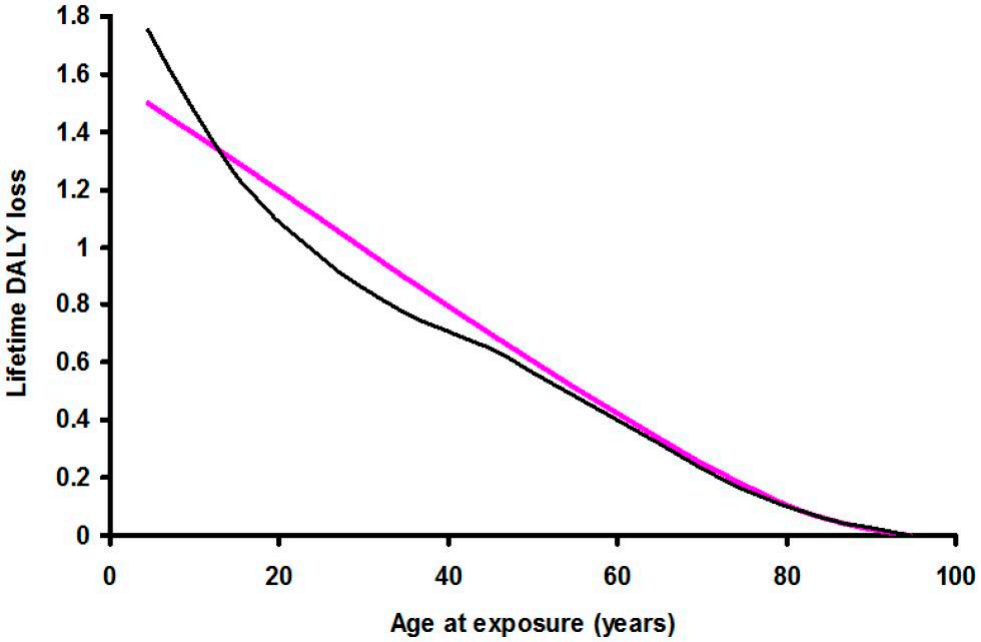
The hypothetical model for individual lifetime loss of disability-adjusted life-years from Figure 3 fitted to the calculated results for a US-European female population using US^13^ and European^14^ life tables to predict the age of death for each point plotted.

**Figure 6.**
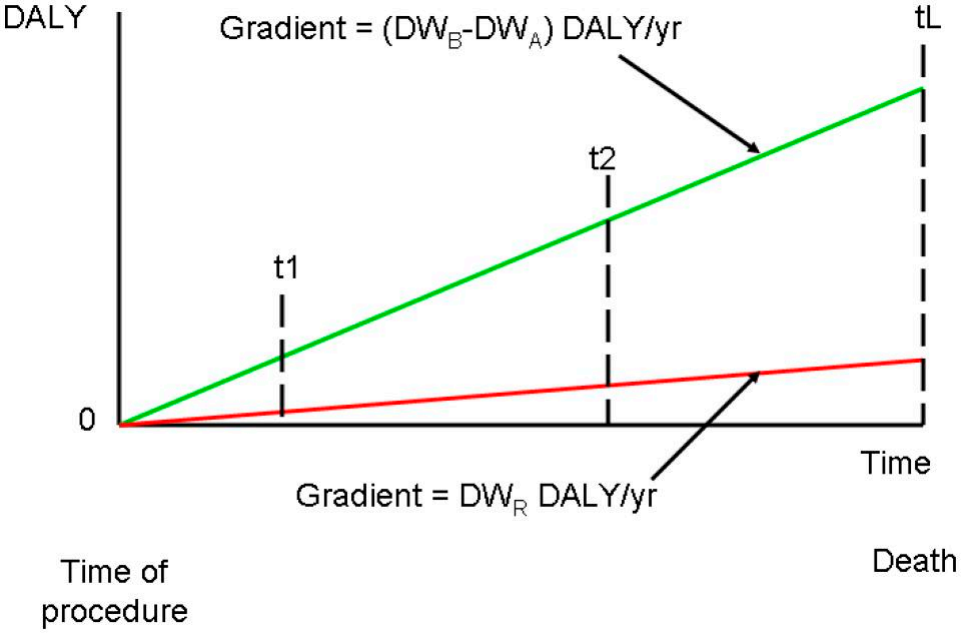
A representation of the time course of medical benefit and radiation detriment in disability-adjusted life-years following a radiological procedure at time zero. The gradient of the benefit (green) line is given the numerator of the justification factor (eqn (3)), and that of the detriment (red) line by the denominator. The ratio of the 2 (J-factor) is constant with time (t1, t2…) from the time of the procedure to death (tL).

**Figure 7.**
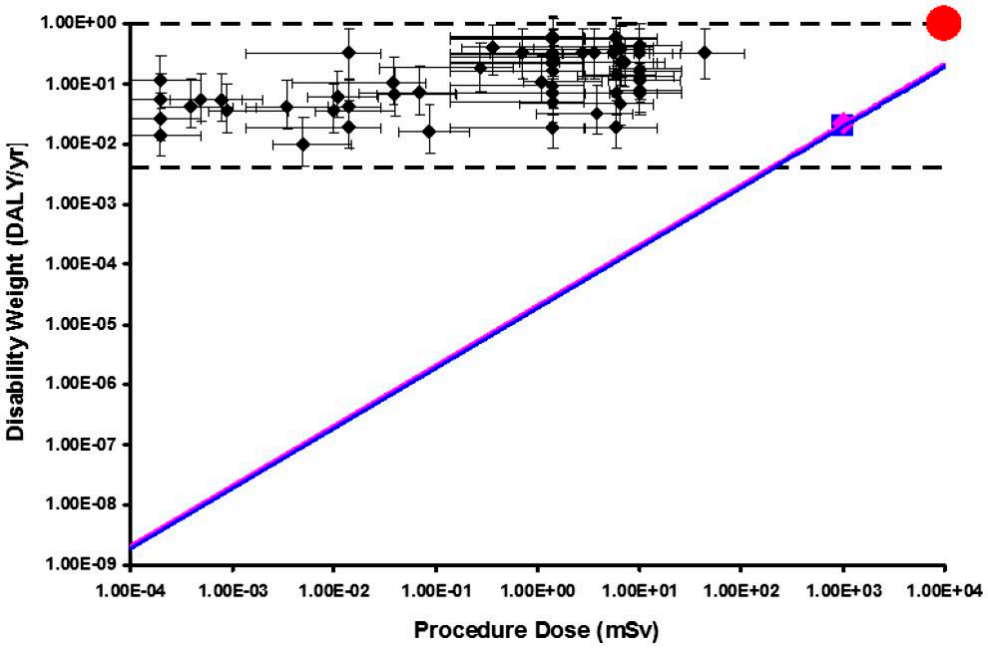
A comparison of DW_B_ with the DW_R_ arising from associated medical radiation exposures. DW_B_ and DW_R_ are plotted on log-log scales of DW (DALY/yr) against total procedure radiation dose in mSv. The dotted lines denote the range of DW_B_ values as defined by WHO.^6^ The large red symbol denotes death from acute radiation syndrome at approximately 10 Sv.^18^ The correspondences between DW_B_ for 66 disease and injury states^6^ commonly associated with radiological examinations (or series of examinations) and their typical total effective doses^19^ are plotted as black diamonds. The pink and blue lines show the values of DW_R_ against dose, for female and male, respectively, with the pink diamond and blue square symbols indicating the values at 1 Sv obtained from the curve fits of Figures 4 and 5.

If the conventional pragmatic linear-no-threshold approach to radiation protection quantities is assumed in line with ICRP Report 103,^3^ then a DW for a radiation exposure of effective dose E can be calculated using the appropriate value of R as:

(2) $$DW_{R}=-R.E$$

where DW_R_ = disability weight due to an exposure from ionizing radiation; R = DALY lost per year due to exposure to 1 Sv of ionizing radiation (male or female); E = effective dose defined in terms of DALY detriment (Sv).

DWs are defined in terms of loss of DALY per year,^6^ so they have positive values and the sign change in eqn (2) is required to fit this definition.

### Application of the disability weight for radiation to the justification of medical exposure

The objective of this work is to establish a link between effective dose, the established measure of the effect of medical exposure to ionizing radiation, and DALY, the established measure of health detriment in populations and the individual in order to quantify the ratio between benefit and detriment for radiological examinations. This ratio can be used as an indicator of justification^15^ in line with the Ionising Radiation (Medical Exposure) Regulations 2017^16^ requirements. Similar international regulations exist.^17^ The results above show that it is actually the rate of change of DALY, the DW in loss of DALY/yr, that scales with effective dose. In a previous paper,^1^ some approaches to estimating lifetime DALY from radiation exposure were explored using data similar to Figures 1 and 2, life tables to estimate remaining lifetime and a known age at exposure. This could still be workable using the curve fits of Figures 4 and 5, but a more attractive idea is to calculate the ratio between the DW beneficially avoided from the successful treatment of a disease or injury, and the detrimental DW_R_ arising from the associated medical radiation exposure. The resulting dimensionless measure is independent of age at exposure on the linear sections of Figures 4 and 5, and is unaffected by the actual life course of the patient after successful treatment. This approach eliminates the requirement to evaluate the years of disability, YD, by assuming that the time from the conclusion of a successful treatment episode to the eventual death of an individual is the same as the time from the associated medical radiation exposure to death. YD is therefore cancelled out in the division of lifetime benefit by lifetime detriment. The logic is that, at the time that a medical exposure is being justified by the medical practitioner, a positive assumption is made that the proposed radiological diagnosis or intervention will be successful and that it will contribute to the overall resolution of the disease or injury being suffered. A successful outcome is that the patient ends the episode of medical care with the short-term and potential lifetime detriment of their disease or injury reduced to zero or a low residual value. If the patient subsequently has their healthy lifetime reduced by related or unrelated disease or injury after the end of the current episode of medical care, then these unknown later events do not affect the current justification decision. Indeed, measures of justification based on statistical life tables would often be erroneous in the case of the individual, and could even be misleading. It seems reasonable, therefore, to consider a justification factor,^1^  *J*, defined as:

(3) $$J=(DW_{B}-DW_{A})/DW_{R}$$

where J = justification factor; DW_B_ = disability weight of disease being diagnosed before treatment; DW_A_ = residual disability weight of disease after treatment; DW_R_ = disability weight due to radiation exposure.

The term (DW_B_ − DW_A_) quantifies the change in DW carried by the individual before and after diagnosis and treatment if the residual DW_A_ is known. For a successful treatment and restoration to full health DW_A_ will be zero. The J-factor expresses the DALY benefit from the successful procedure divided by the DALY detriment due to the associated medical radiation exposure in any time period following successful treatment, regardless of the actual eventual lifespan of the individual patient. This is illustrated in Figure 6 which shows the accumulation of DALY benefit from the successful procedure (green line) and DALY detriment from the associated radiation exposure (red line). At the time of the patient’s eventual death, the lifetime ratio of DALY benefit to detriment will be the value at the end of the green line divided by that at the end of the green line. This ratio is, however, constant at all times, t1, t2, etc so the justification factor (eqn (3)) applies in any time scale following the successful procedure.

## Results

Figure 7 compares DW_B_ for 66 common medical conditions with the DW_R_ arising from associated medical radiation exposures. The figure plots values of DW_B_ and DW_R_ on log-log scales of DW (DALY/yr) against total procedure radiation dose in mSv. The horizontal dotted lines denote the complete range of DW_B_ values as defined by WHO,^6^ which extend from 0.004 for mild anaemia (described as “feels slightly tired and weak at times, but this does not interfere with normal daily activities”^6^) to 1.0 for death. The extent of the diagram is limited by the large red symbol in the top right, which denotes death from acute radiation syndrome at approximately 10 Sv.^18^ The correspondences between DW_B_ for 66 disease and injury states^6^ commonly associated with radiological examinations (or series of examinations) and their typical total effective doses^19^ are plotted as black diamonds. Uncertainty limits on DW_B_ are calculated from the study of Lopez et al,^20^ and those on effective dose from the study of Hart et al.^19^ The pink and blue lines show the values of DW_R_ against dose, for female and male, respectively, with the pink diamond and blue square symbols indicating the values at 1 Sv obtained from the curve fits of Figures 4 and 5. The slope of these lines on log-log scales is fixed by the assumption of a linear-no-threshold relationship between dose and radiation detriment.

It is clear from the diagram that the values of DW_B_ are higher, usually much higher, than the associated DW_R_ for all the radiological procedures plotted (which range from dental radiography through CT to interventional procedures), so all of the associated justification factors (eqn (3)) will be much greater than unity for an assumed fully successful treatment when the resulting value of DW_A_ will be zero. All of these procedures will therefore be justified in terms of benefit exceeding detriment. Indeed, it is difficult to see how any clinically indicated radiological procedure could fail to be justified on this basis as it would have to have a dose in excess of 200 mSv combined with a DW_B_ associated with a very low impact on the health of the patient. Even if the derivation above has led to inaccurately low estimates for DW_R_, it is equally difficult to envisage how they could logically be much higher without overtaking at high doses the detriment of acute radiation syndrome, which includes many additional tissue effects over and above the cancers included in DW_R_.

## Discussion

Figure 7 gives a general overview of the relationship between the DWs for cases of disease and injury, DW_B_ and the DWs for medical radiation exposure associated with those conditions, DW_R_. Whilst this is a simple way of quantifying and demonstrating overall benefit and detriment for a wide range of radiological procedures, some underlying assumptions need to be examined.

Although the detriment element, DW_R_, contains within it the detriment from premature mortality as well as morbidity (YLL + YLD in eqn (1), total lifetime DALY in Figures 1 and 2), the benefit element, DW_B_ is just the prevailing DW from the YLD part of DALY. Disease and injury mortality is therefore not part of the comparison, under the assumption that most routine radiological examinations are associated with non-fatal conditions. Even though the justification factors, J (eqn (2)) are already consistently high, they would be even higher for the case of a radiological intervention resulting in the avoidance of death. If this was genuinely the case then a DW_B_ value of 1.0 could be used without changing the definitions of eqn (3).

Where the condition to be diagnosed and treated involves a series of radiation exposures, these have been summed to illustrate the highest dose, lowest J-factor case.^1^ No allowance has been made for missed or incorrect radiological findings, or for unsuccessful treatments, as the expectation of the practitioner at the time of the justification decision is one of a successful outcome.^1^

It is important that Figure 7 is not interpreted as a reason to relax dose optimization. The large implied justification factor values for the various examinations and procedures can instead be taken as reflective of the degree of optimization already achieved. It would be counter to the principles of optimization and against UK regulations^16^ to take any action which intentionally reduces these values.

## Conclusions

The health detriment, defined as lifetime loss of DALY at age of exposure to ionizing radiation for a US-European population has been calculated and a simple model of the relationship fitted to the results. Apart from in late life within the latency period for radiation-induced cancers, most of the relationship can be adequately fitted to a straight line of negative gradient. The gradient of this line corresponds to a loss of DALY per year following exposure to radiation and is therefore equivalent to a DW used in the calculation of DALY. This definition of a DW for ionizing radiation, proportional to effective dose as currently defined, can link radiation exposure to the existing large body of data on the DALY burden and DWs for a wide range of diseases and injuries, providing a means for the quantitative justification of medical exposures. A graphical analysis of the relationship between medical benefit and radiation detriment for a wide range of radiological procedures strongly suggests that, with the current state of dose optimization, all procedures that are justified as being advantageous for clinical management will also be justified in terms of the ratio of potential health benefit to radiation detriment.
